# A new way to regulate inflammation: selective autophagic degradation of IKKγ mediated by ANGPTL8

**DOI:** 10.15698/cst2018.03.128

**Published:** 2018-02-14

**Authors:** Yu Zhang, Ling Zheng, Kun Huang

**Affiliations:** 1 Tongji School of Pharmacy, Tongji Medical College, Huazhong University of Science & Technology, Wuhan, P.R.China, 430030.; 2 Hubei Key Laboratory of Cell Homeostasis, College of Life Sciences, Wuhan University, Wuhan, P.R.China, 430072.

**Keywords:** NF-κB, ANGPTL8, signal transduction, oligomerization, selective autophagy

## Abstract

Transcription factor nuclear factor-κB (NF-κB) plays pivotal roles in the regulation of inflammation and immunity. However, the precise mechanism of NF-κB activation is not fully elucidated. We recently found that Angiopoietin like protein 8 (ANGPTL8, also known as Lipasin, RIFL, TD26 or C19orf80), which is a previously identified secreted metabolic regulator, also can work intracellularly as a negative feedback inhibitor of NF-κB activation. Mechanistically, ANGPTL8 is induced by TNFα stimulation, a classic inducer for NF-κB activation; then, the intracellular ANGPTL8 self-associates via its N-terminal region and interacts with Sequestosome-1 (p62/SQSTM1). The resulting ANGPTL8-p62 aggregates work as a platform in the recruitment and autophagic degradation of IκB kinase gamma (IKKγ/NEMO). Consistently, in patients diagnosed with infectious diseases, enhanced circulating ANGPTL8 levels were detected. These findings suggest a new role for selective autophagy in the regulation of signal transduction and inflammation.

Activation of transcription factor nuclear factor-κB (NF-κB) is one of the central events in inflammation and immunity. In resting cells, the activity of NF-κB is inhibited by its binding partner, inhibitor of κB α (IκBα). The IκBα kinase (IKK) complex controls the phosphorylation of IκBα to ensure its proper degradation, a process that releases NF-κB into the nucleus to turn on its target genes. IKK complex consists of catalytic subunits IKKα/β that phosphorylate IκBα, and a regulatory subunit IKKγ which mediates the IKK complex formation and signal transduction. Since excessive NF-κB activation plays critical roles in many autoimmune and inflammatory diseases, the precise mechanism behind restriction of NF-κB signaling is of great interest.

Generally, signaling pathways may be down-regulated through three related mechanisms - (i) inactivation of signaling components via specific protein modifications such as ubiquitination and dephosphorylation, (ii) disassociation of the signal complexes, and (iii) proteolysis of signal molecules by the ubiquitin-proteasome system (UPS), the latter being the most commonly seen mechanism. Another important route to mediate protein degradation is autophagy, which involves the sequestration of intracellular material in double-membrane vesicles, termed autophagosomes, and their digestion by fusion with lysosomes. It has been thought that the UPS is a selective way to regulate proper signaling, whereas autophagy is a bulk process that nonspecifically responds to multiple stresses. This concept has been partly changed after the recent discovery of selective autophagy, in which K63-polyubiquitinated substrates are specifically transferred to autophagic receptors such as p62, and degradated in autophagosomes. So far, selective autophagy has been known to mediate the degradation of damaged or senescent cell organelles, invasive microbes, misfolded proteins or aggregates; yet, its function in the degradation of signaling molecules has remained unclear.

Our recent study (Nature Communications, 8: 2164) identified ANGPTL8 as a new inhibitor in NF-κB activation by mediating the selective autophagic degradation of IKKγ. Upon inflammatory stimuli such as TNFα treatment, ANGPTL8 is induced which facilitates the degradation of IKKγ specifically. Genetic or chemical blockage of autophagosomes, but not of the proteasome, inhibits ANGPTL8-facilitated IKKγ degradation. Furthermore, ANGPTL8 works as a "co-receptor" of p62 by facilitating the formation of a p62-ANGPTL8-IKKγ complex, thereby enhancing the autophagic IKKγ degradation upon TNFα treatment. The N-terminal region (residues 26-70) mediates the self-association of ANGPTL8, and the resulting ANGPTL8 oligomers play a critical role in the IKKγ degradation and NF-κB inactivation (**Figure 1**).

**Figure 1:**
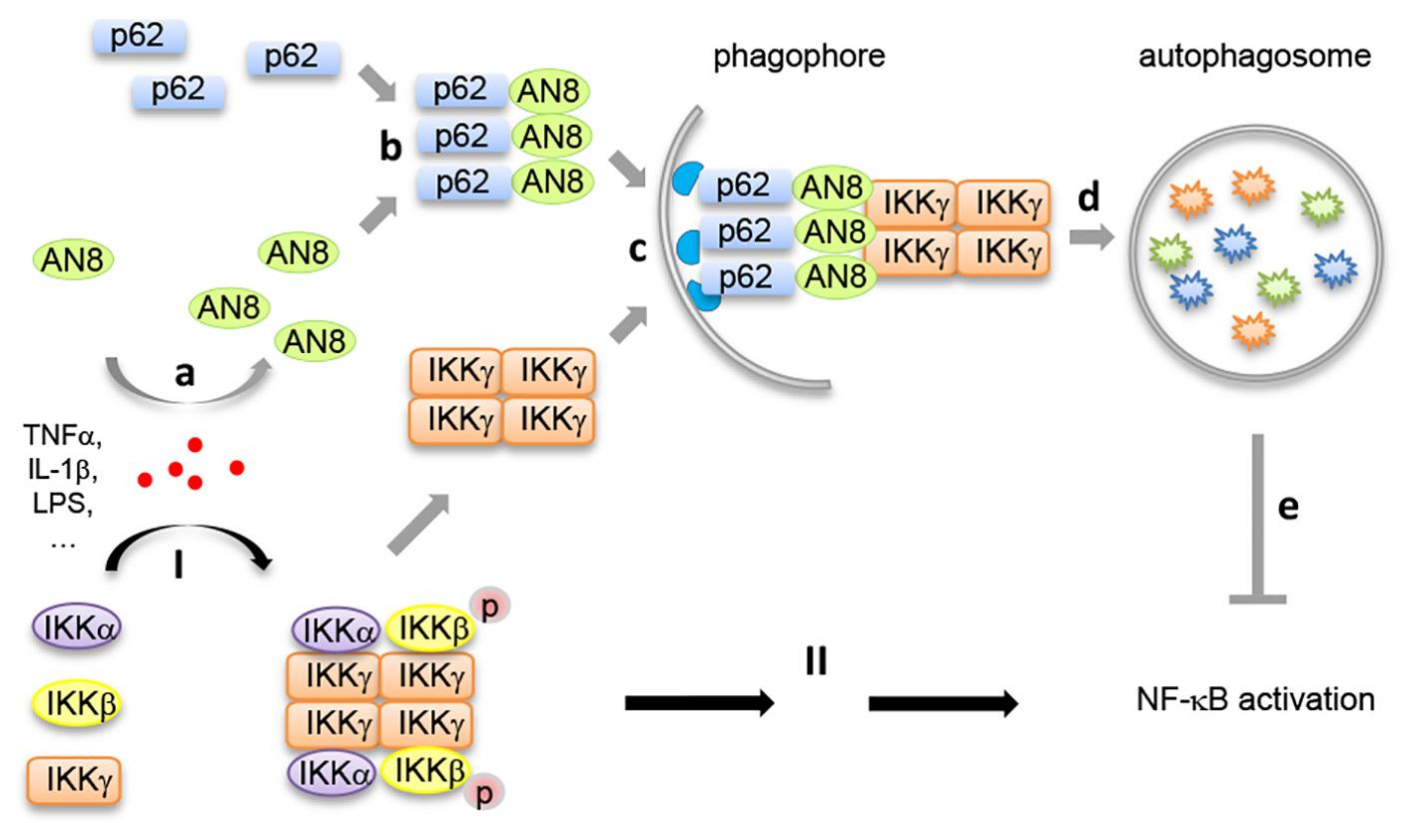
FIGURE 1: A negative feedback model of how self-associated ANGPTL8 facilitates autophagic IKKγ degradation. Upon TNFα stimulation, the IKK complexes are formed and activated through the oligomerization of IKKγ (**I**), to ensure proper NF-κB activation (**II**). At the same time, inflammatory stimuli such as TNFα induces the up-regulation of ANGPTL8 (AN8, **a**), then ANGPTL8 and p62 form hetero-oligomers (**b**), self-association of ANGPTL8 mediates the p62-IKKγ recognition (**c**); finally, the ANGPTL8-p62-IKKγ complex is degraded in autophagosomes (**d**). This negative feedback process contributes to the precision control of the NF-κB activation (**e**).

It has been demonstrated that the oligomerization of autophagy receptor proteins (such as p62) is the precondition for substrate recognition and separation. We reported for the first time that ANGPTL8 self-associates, and that this oligomerization, in conjunction with p62 binding, is essential for its interaction with IKKγ and the subsequent degradation of IKKγ. Moreover, our work indicated that the coiled-coil domain of ANGPTL8 (residues 71-198) directly interacts with p62, while the self-association region of ANGPTL8 (residues 26-70) is responsible for the formation of heteromultimers which recognize aggregated IKKγ **(Figure 1)**. Interestingly, while there are numerous substrates that await degradation, there are less than ten autophagy receptors identified, which raises a key scientific question how autophagy receptors can recognize the right substrate(s) at the right condition. Our study may partly answer this question, that is, in addition to the few known autophagy receptors, there may exist many additional "co-receptors" that facilitate the cargo recognition and degradation. Since selective autophagy often degrades "giant" cargoes, receptor and co-receptor may need to interact with each other to form large hetero-oligomers to accomplish such a difficult mission.

The preference of selective autophagy in mediating the degradation of "huge" cargoes makes aggregation a prerequisite for protein substrates. Structurally, there are two types of protein aggregation, mediated by α-helix and β-sheet, respectively. It is generally accepted that the former one represents physiological aggregation in signal transduction, while the latter is usually involved in the pathological process such as neurodegeneration. Many studies have suggested that β-sheet-mediated misfolded proteins or aggregates are suitable substrates for selective autophagy. IKKγ is a scaffold protein, whose α-helix-based oligomerization is essential for signaling transduction; however, our study indicates that the oligomerization of IKKγ is not only critical for its physiological functions, but also acts as a signal that mediates its degradation. Therefore, our study indicates selective autophagy as part of a precisely orchestrated machinery that finely controls signal transduction, despite the "huge" cargoes of selective autophagy.

Physiologically, in mice challenged with LPS, we found that tissues with higher mRNA level of Angptl8 are more sensitive to inflammatory stress and exhibit a faster decline of transcription of NF-κB target genes, which implicates that ANGPTL8 may function as a "brake" for inflammation. We also noted markedly enhanced circulating ANGPTL8 in the patients with infectious disease. These results suggest that secreted ANGPTL8 may be involved in the regulation of inflammation, in addition to its previously reported regulatory role in lipid metabolism. Since the self-association region of ANGPTL8 (residues 26-70), which are essential for ANGPTL8-mediated inflammatory regulation, is present in both secreted and intracellular form of ANGPTL8, we surmise that self-association may also play physiological roles in circulating ANGPTL8.

ANGPTL8 is a member of the ANGPTL family, consisting of eight structurally similar proteins, named ANGPTL1-8, which play roles in the regulation of lipid and glucose metabolism, hematopoiesis, and cancer. Members of ANGPTLs have been shown to work cooperatively. For example, ANGPTL3-4-8 could form a complex to regulate the dynamics of triglyceride trafficking under fasting and feeding conditions. It will be interesting to investigate that whether other ANGPTL8 family members also play roles in mediating inflammation such as NF-κB activation.

In summary, our study identifies a secreted metabolic regulator ANGPTL8 as a negative feedback regulator in the control of fine-tuned inflammation. This study broadens our understanding of selective autophagy, and provides insights to the comprehension of the crosstalk of metabolism and inflammation.

